# Supplementary material for: The RNAi Pathway Is Important to Control Mayaro Virus Infection in Aedes aegypti but not for Wolbachia-Mediated Protection
Source: Viruses. 2020 Aug 10;12(8):871. doi: 10.3390/v12080871 (PMC7547387; doi:10.3390/v12080871)
Supplement: Supplementary file 1 [file viruses-12-00871-s001.pdf]

viruses

Supplementary Materials: The RNAi pathway is important to control Mayaro virus infection in *Aedes aegypti* but not for *Wolbachia*-mediated protection

Table S1. Oligonucleotides sequences.

Target	Description	Sequence (5′–3′)	Application	Reference
RPL32 AAEL003396	RpL32 Fw	AGCCGCGTGTTGTACTCTG	qPCR	[36]
	RpL32 Rv	ACTTCTTCGTCCGCTTCTTG		
MAYV	MAYV Fw	GTGGTCGCACAGTGAATCTTTC	qPCR	[10]
	MAYV Rv	CAAATGTCCACCAGGCGAAG		
Firefly luciferase U47295	FLUC Fw (T7)	taatacgactcactatagggagaAACAATCCGGAAGCGACCAA	dsRNA synthesis	[36]
	FLUC Rv (SP6)	atttaggtgacactatagaagtgTGACTGGCGACGTAATCCAC		
AGO2 AAEL017251	AGO2 Fw	AGATTGACAAGCAGAAAATCCAC	qPCR	
	AGO2 Rv	CATTTGGACGCATCAGCA		
	AGO2 Fw (T7)	taatacgactcactatagggagaCAGTTCAAGCAGACGAACCA	dsRNA synthesis	
	AGO2 Rv (SP6)	atttaggtgacactatagaagTGATGTAGACGCGTCCTCTG		

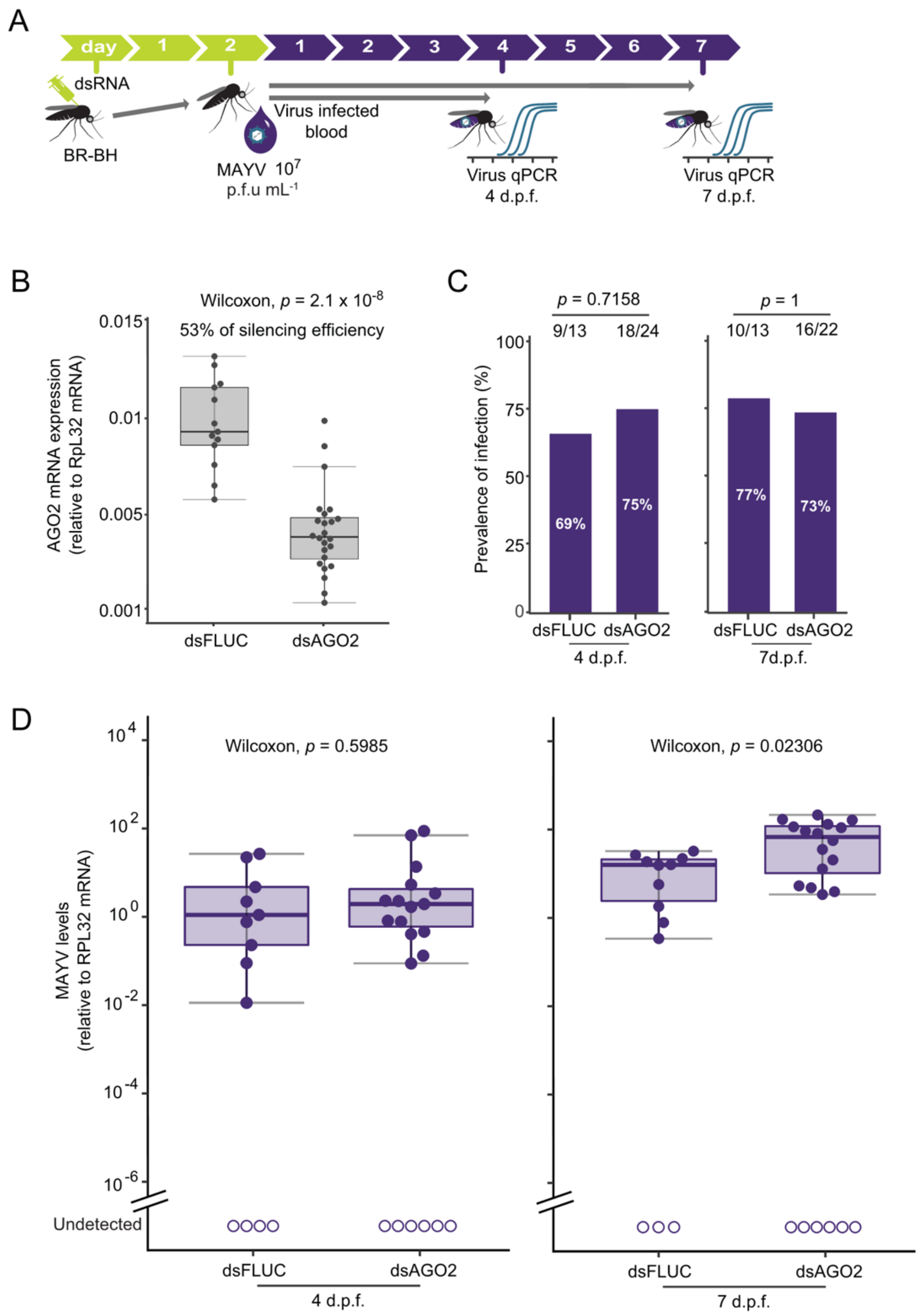

Figure S1. The effect of siRNA pathway in *Aedes aegypti* infected with lower dose of MAY (10^7 p.f.u. ml^{-1}). (**A**) Scheme of the silencing process using dsRNA. BR-BH females were intrathoracically injected with dsAGO2 and dsFLUC (control group). 2 days post microinjection, mosquitoes were fed on a blood meal containing 10^7 p.f.u. ml^{-1}of MAYV. Mosquitoes were collected at 4 and 7 d.p.f and tested individually by qPCR to detect viral RNA levels. (**B**) Mosquitoes collected at 4 d.p.f were also tested individually by qPCR for AGO2 mRNA expression for measuring the silencing efficiency. Each dot represents an individual whole mosquito. Statistical analyses were performed using the Mann-Whitney-Wilcoxon test, comparing the Ago2 expression levels. (**C**) Prevalence of infection. Total number of mosquitoes tested are indicated above each column. Statistical analyses were performed using the two-tailed Fisher's exact test. (**D**) MAYV RNA levels at 4 and 7d.p.f. Each dot represents an individual whole mosquito. Statistical analyses were performed using the Mann-Whitney-Wilcoxon test, comparing the MAYV titers of the infected mosquitoes.

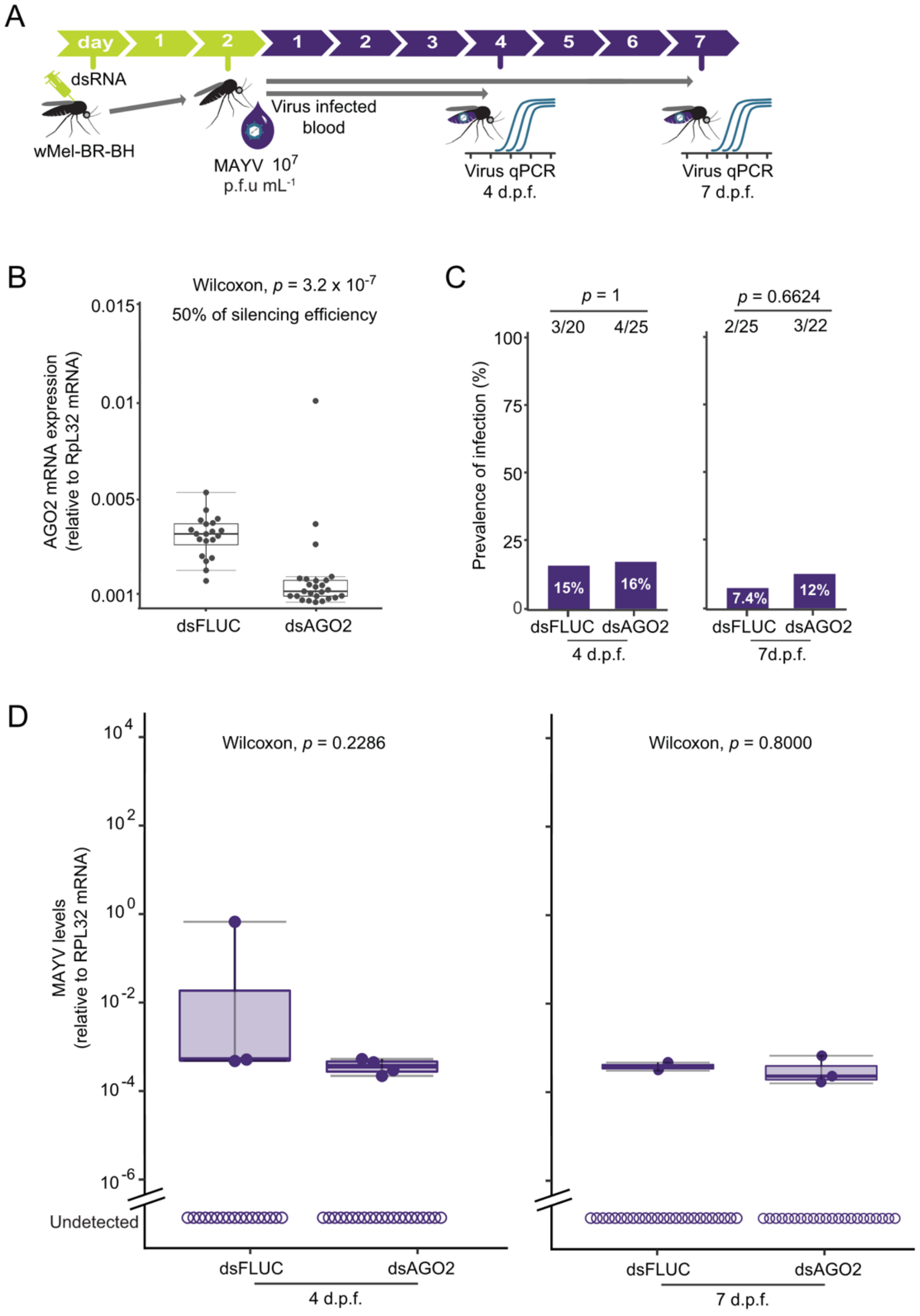

Figure S2. The effect of the mosquito siRNA pathway on *Wolbachia*-mediated MAYV protection using a lower dose of MAY (10^7 p.f.u. ml^{-1}). (**A**) Scheme of the silencing process using dsRNA. *w*Mel-BR-BH females were intrathoracically injected with dsAGO2 and dsFLUC (control group). 2 days post microinjection, mosquitoes were fed on a blood meal containing 10^7 p.f.u. ml-1 of MAYV. Mosquitoes were collected at 4 and 7 d.p.f and tested individually by qPCR to detect viral RNA levels. (**B**) Mosquitoes collected at 4 d.p.f were also tested individually by qPCR for AGO2 mRNA expression for measuring the silencing efficiency. Each dot represents an individual whole mosquito. Statistical analyses were performed using the Mann-Whitney-Wilcoxon test, comparing the AGO2 expression levels. (**C**) Prevalence of infection. Total number of mosquitoes tested are indicated above each column. Statistical analyses were performed using the two-tailed Fisher's exact test. (**D**) MAYV RNA levels at 4 and 7d.p.f. Each dot represents an individual whole mosquito. Statistical analyses were performed using the Mann-Whitney-Wilcoxon test, comparing the MAYV titers of the infected mosquitoes.
